# Identification and mechanistic investigation of novel biomarkers for diabetic retinopathy through hypoxia- and cuproptosis-associated gene analysis

**DOI:** 10.1371/journal.pone.0358182

**Published:** 2026-09-25

**Authors:** Rong Feng, Xia Wang, Xiaodong Li, Zhifang Zhao, Bendan Long, Hui Zhang, Fengjuan Yue

**Affiliations:** 1 Health Examination Center, Guizhou Provincial People’s Hospital, Guiyang, Guizhou, China; 2 Gastroenterology Department, Guizhou Provincial People’s Hospital, Guiyang, Guizhou, China; 3 Endocrinology Department, Guizhou Provincial People’s Hospital, Guiyang, Guizhou, China; Huazhong Agriculture University, CHINA

## Abstract

**Background:**

Emerging evidence indicates that hypoxia- and cuproptosis-related molecular alterations may contribute to diabetic retinopathy (DR). This study aimed to identify biomarkers associated with these processes and investigate their biological relevance in DR.

**Methods:**

The DR-related datasets GSE189005 and GSE221521 were sourced from public databases, while hypoxia-related and cuproptosis-related genes were extracted from published studies. Genes consistently differentially expressed across both datasets were intersected with those associated with hypoxia- and cuproptosis-related expression scores to derive candidate genes. Selection of candidate biomarkers was performed using machine-learning algorithms and expression consistency analysis, with diagnostic performance assessed via receiver operating characteristic (ROC) curves. Functional enrichment, immune cell infiltration, regulatory network analysis, candidate compound prediction, and reverse transcription quantitative PCR (RT-qPCR) were also conducted.

**Results:**

Intersection screening yielded 13 candidate genes. Machine-learning analysis identified three candidate biomarkers. Notably, MED19 and CA11 were consistently downregulated in DR, exhibiting area under the curve (AUC) values exceeding 0.70 in both datasets, thus qualifying as biomarkers. Functional enrichment analysis suggested MED19's involvement in “proteasome” and “spliceosome” pathways, while CA11 was linked to “regulation of autophagy” and “basal cell carcinoma” pathways. Differential infiltration of three immune cell populations (eosinophils, M2 macrophages, activated natural killer cells) was noted between DR and control groups in GSE189005, with M2 macrophages demonstrating a significant negative correlation with both biomarkers. Regulatory network analysis highlighted several candidate transcriptional regulators for MED19 and CA11, including ELF1–MED19 and KDM5B–CA11. Thirty candidate compounds/interventions were identified, including schizandrin B and pirinixic acid. RT-qPCR confirmed the significant downregulation of MED19 and CA11 in DR samples, providing preliminary support.

**Conclusion:**

MED19 and CA11 were identified as biomarkers, presenting a potential framework for therapeutic intervention in DR.

## 1. Introduction

Diabetes mellitus frequently leads to diabetic retinopathy (DR), a microvascular disorder characterized by progressive degeneration of retinal blood vessels, which can result in visual impairment or complete blindness if untreated [1,2]. DR progresses through two stages: non-proliferative DR (NPDR) and proliferative DR (PDR). NPDR is marked by microaneurysms, retinal hemorrhages, and hard exudates, while progression to PDR involves altered retinal cellular function and pathological neovascularization, potentially leading to retinal detachment and severe vision loss [3]. Approximately 50% of patients with PDR experience total vision loss within five years, imposing significant economic burdens on families, healthcare systems, and society [4]. Early DR is often asymptomatic, with the onset of symptoms indicating advanced, irreversible disease, where interventions can only slow its progression [5]. Consequently, the identification of novel, reliable, and effective biomarkers is critical for early detection and targeted therapy, potentially enhancing patient outcomes.

Persistent hyperglycemia and systemic metabolic disturbances contribute to the development of diabetic complications through oxidative stress, inflammation, abnormal insulin signaling, and metabolic damage. Recent reviews on bioactive substances, including cycloether terpenoids and edible mushroom polysaccharides, suggest that glucose homeostasis, inflammatory signaling, and oxidative stress are interconnected in diabetes-related diseases. In the retina, these systemic metabolic abnormalities may exacerbate microvascular dysfunction and oxidative damage, resulting in a local hypoxic microenvironment. Consequently, hypoxia is a key pathological feature of DR [6,7]. Hypoxic retinal tissues activate hypoxia-inducible factor-1α (HIF-1α)-related signaling pathways and enhance the expression of vascular endothelial growth factor (VEGF) [8,9]. Additionally, cuproptosis, a recently characterized mechanism of cell death, has been implicated in DR [10]. Evidence suggests that hypoxia may alter copper ion levels, potentially acting synergistically with VEGF to co-activate HIF-1α expression, thereby influencing retinal angiogenesis [9]. These findings indicate that modulating retinal hypoxia and altered cuproptosis responses *in vivo* could impact VEGF and HIF-1α expression, potentially slowing the progression of DR, although further validation is necessary. While hypoxia-related genes (HRGs) and cuproptosis-related genes(CRGs) have been individually associated with DR, no prior study has integrated HRG and CRG expression signatures to identify co-regulated biomarkers in DR blood transcriptomes. This integrative approach may uncover synergistic molecular interactions overlooked by single-pathway analyses. Accordingly, this study aimed to identify DR biomarkers associated with both hypoxia and cuproptosis and to explore their potential mechanistic roles.

**Fig 1:**
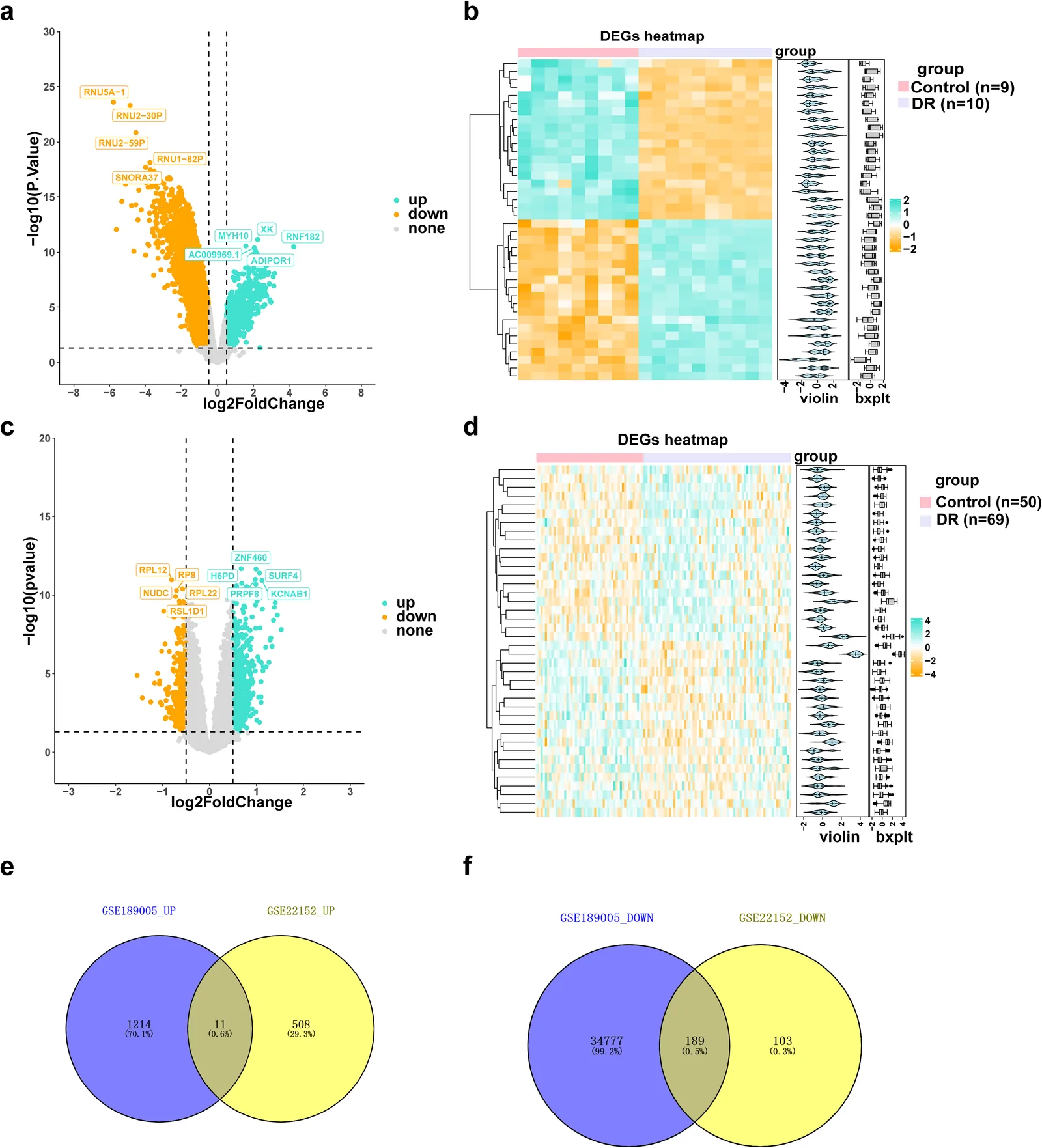
Overview of the study workflow.

In this study, resources from the Gene Expression Omnibus (GEO) database were utilized to screen for biomarkers through differential expression analysis, machine learning algorithms, and gene expression profiling, with diagnostic performance assessed using ROC analysis. Subsequent analyses included biomarker functional enrichment, regulatory network interactions, and immune cell correlations, aiming to provide novel insights for the treatment of DR. The specific analysis workflow is illustrated in Fig 1.

## 2. Materials and methods

### 2.1. Data acquisition

Transcriptomic datasets related to DR, specifically GSE189005 and GSE221521, were retrieved from the GEO database (https://www.ncbi.nlm.nih.gov/geo/). GSE189005 comprised 10 DR and 9 control blood samples using the GPL23126 platform, while GSE221521 included 69 DR and 50 control blood samples on the GPL24676 platform. GSE189005 was normalized using the RMA method (background correction, log_2_ transformation, quantile normalization), whereas GSE221521 was normalized with DESeq2 median-of-ratios. Datasets were preprocessed separately to minimize cross-platform batch effects, and no cross-dataset correction was performed. PCA analysis (R prcomp) confirmed clear clustering without outliers or batch effects in either dataset (S1 Fig). Additionally, 254 HRGs [11] and 19 CRGs [12] were obtained from the literature.

### 2.2. Differential expression analysis for GSE189005 and GSE221521

Differential expression analysis was conducted separately for GSE189005 and GSE221521 using the “limma” package (v3.52.4) [13]. Genes meeting the criteria of |log_2_FC| > 0.5 and nominal *p* < 0.05 were classified as DR-associated differentially expressed genes in each dataset. During the preliminary screening, a relatively lenient nominal P-value threshold was applied to enhance sensitivity and prevent premature exclusion of potentially relevant features. Volcano plots and heatmaps (top 40 genes by |log_2_FC|) were generated using “ggplot2” (v3.3.6) [14] and “pheatmap” (v1.1.9), respectively. Upregulated and downregulated genes were intersected separately across the two datasets, defining genes exhibiting the same direction of differential expression as consistently differentially expressed DR genes.

### 2.3. Identification of candidate genes

In GSE189005, hypoxia- and cuproptosis-related expression scores were calculated separately using “GSVA” (v1.1.9) [15] based on the respective sets of 254 HRGs and 19 CRGs. Samples were categorized into high- and low-score groups according to the median score, and differential expression analysis was performed between the two groups. The resulting gene sets were defined as hypoxia-score-associated genes and cuproptosis-score-associated genes, respectively. Visualization of results included volcano plots and heatmaps. Genes shared among the consistently differentially expressed DR genes, hypoxia-score-associated genes, and cuproptosis-score-associated genes were defined as candidate genes. This intersection strategy was employed to retain genes consistently differentially expressed in DR across both datasets and associated with hypoxia- and cuproptosis-related expression patterns.

### 2.4. Machine learning implementation

Candidate biomarker selection was performed on the candidate genes using the Least Absolute Shrinkage and Selection Operator (LASSO) and Support Vector Machine-Recursive Feature Elimination (SVM-RFE) methods. LASSO analysis utilized the “glmnet” package (v4.1-4) with family = “binomial” and three-fold cross-validation, selecting features at the λ value minimizing deviance (lambda.min) [16]. SVM-RFE was executed using “e1071” (v1.7-13) with three-fold cross-validation, iteratively removing features (k = 3) based on model error [17]. Final candidate biomarkers were identified as the intersection of features selected by LASSO and SVM-RFE.

### 2.5. Biomarker identification and validation

For biomarker identification, gene expression analysis of candidate biomarkers was conducted using the “ggplot2” package in GSE189005 and GSE221521. Biomarkers were selected based on significant differential expression between DR and control samples (*p* < 0.05) with consistent expression trends across both datasets for further analysis.

The predictive capability was assessed by constructing ROC curves for each dataset to evaluate diagnostic potential, with genes showing an area under the curve (AUC) ≥ 0.7 considered to exhibit adequate diagnostic effectiveness for DR.

The GeneMANIA database (http://genemania.org/) facilitated exploration of potential interactions and functional relationships between biomarkers and other genes, allowing the identification of associated genes and the construction of gene-gene interaction (GGI) networks.

Spearman's rank correlation analysis was performed using the “psych” R package (v2.2.9) on the GSE189005 dataset to evaluate associations between identified biomarkers and hypoxia/cuproptosis markers. Expression levels were compared against hypoxia markers (HIF1A, VEGFA) and cuproptosis markers (FDX1, LIAS), with significant correlations defined as |r| > 0.3 and *p* < 0.05.

### 2.6. Gene set enrichment analysis (GSEA)

Functional annotation of biomarkers was conducted through correlation profiling within the GSE189005 transcriptome, ranking genes by descending correlation coefficients. KEGG gene sets from the “org” package (v3.15.0) [18] served as the background for GSEA analysis performed using the “clusterProfiler” package (v4.4.4) [19], applying thresholds of |NES| > 1, *p* < 0.05, and q < 0.25.

### 2.7. Immune infiltration assessment

Immune cell infiltration was quantified using CIBERSORT to assess 22 immune cell populations in DR and control samples from GSE189005. Immune cells with zero abundance were excluded, and results were visualized via heatmaps. Differences between DR and control groups were evaluated using Wilcoxon rank-sum tests (*p* < 0.05), while associations between biomarkers and immune cells were analyzed using Spearman correlation (|ρ| > 0.30, *p* < 0.05) and visualized with “ggcorrplot” (v0.92) [20]. Additionally, Spearman correlation analysis between MED19/CA11 and immune regulators (IL10, CCL2, CCL22, PDCD1, CTLA4, LAG3, HAVCR2, CD274) was performed using GSE189005, with results visualized as a heatmap.

### 2.8. Regulatory network analysis

Transcription factors (TFs) associated with biomarkers were predicted using ChIP-Enrichment Analysis version 3 (ChEA3, https://amp.pharm.mssm.edu/chea3/). The top 20 TFs were selected to construct a TF-mRNA regulatory network, which was visualized using “Cytoscape” (v3.10.0) [21].

### 2.9. Candidate compound prediction

The Comparative Toxicogenomics Database (CTD, https://ctdbase.org/) was employed to identify candidate compounds targeting biomarkers.Because CTD-based compound-gene associations do not by themselves demonstrate therapeutic efficacy or reversal of disease-associated gene expression, a secondary prioritization was performed according to available toxicity information, endocrine-disrupting potential, and ophthalmology-related evidence. Candidates were assigned to Tier 1 if they had no reported endocrine disruption and had ophthalmology-related research evidence, Tier 2 if they had no reported endocrine disruption but lacked ophthalmology-related evidence, and Tier 3 if they were known or suspected endocrine disruptors or had other documented toxicities. Broad exposure categories without a defined molecular entity were not tiered. Candidate compound-biomarker networks were constructed and visualized with “Cytoscape.”

### 2.10. Reverse transcription quantitative PCR (RT-qPCR)

Ten blood samples (5 normal and 5 from patients with DR) were collected from the Guizhou Provincial People's Hospital clinic, reflecting the exploratory nature of this pilot study and the limited availability of samples; larger studies are needed for validation. Detailed clinical information is provided in S1 Table. The study protocol received approval from the Institutional Review Board of Guizhou Provincial People's Hospital (NO.2025−188, Sep 22, 2025), and written informed consent was obtained from all participants. Total RNA was extracted using TRIzol reagent (Ambion, USA) according to the manufacturer's instructions. RNA concentration was assessed with a NanoPhotometer N50. cDNA was synthesized using the SureScript First-strand cDNA Synthesis Kit, with reverse transcription performed on a S1000™ Thermal Cycler (Bio-Rad, USA). All primer sequences are listed in S2 Table. qPCR was conducted on a CFX Connect Real-time Quantitative Fluorescence PCR Instrument (Bio-Rad, USA) under the following conditions: initial denaturation at 95℃ for 1 minute, followed by denaturation at 95℃ for 20 seconds, annealing at 55℃ for 20 seconds, and extension at 72℃ for 30 seconds, for a total of 40 cycles. Relative mRNA levels were calculated using the 2^-ΔΔCT^ method. RT-qPCR results were exported to Excel and imported into GraphPad Prism 5 for statistical analysis and visualization.

### 2.11. Statistical analysis

All statistical analyses were performed using R (version 4.2.3), with *p* < 0.05 considered statistically significant unless otherwise indicated.

## 3. Results

### 3.1. Identification of 13 candidate genes

**Fig 2:**
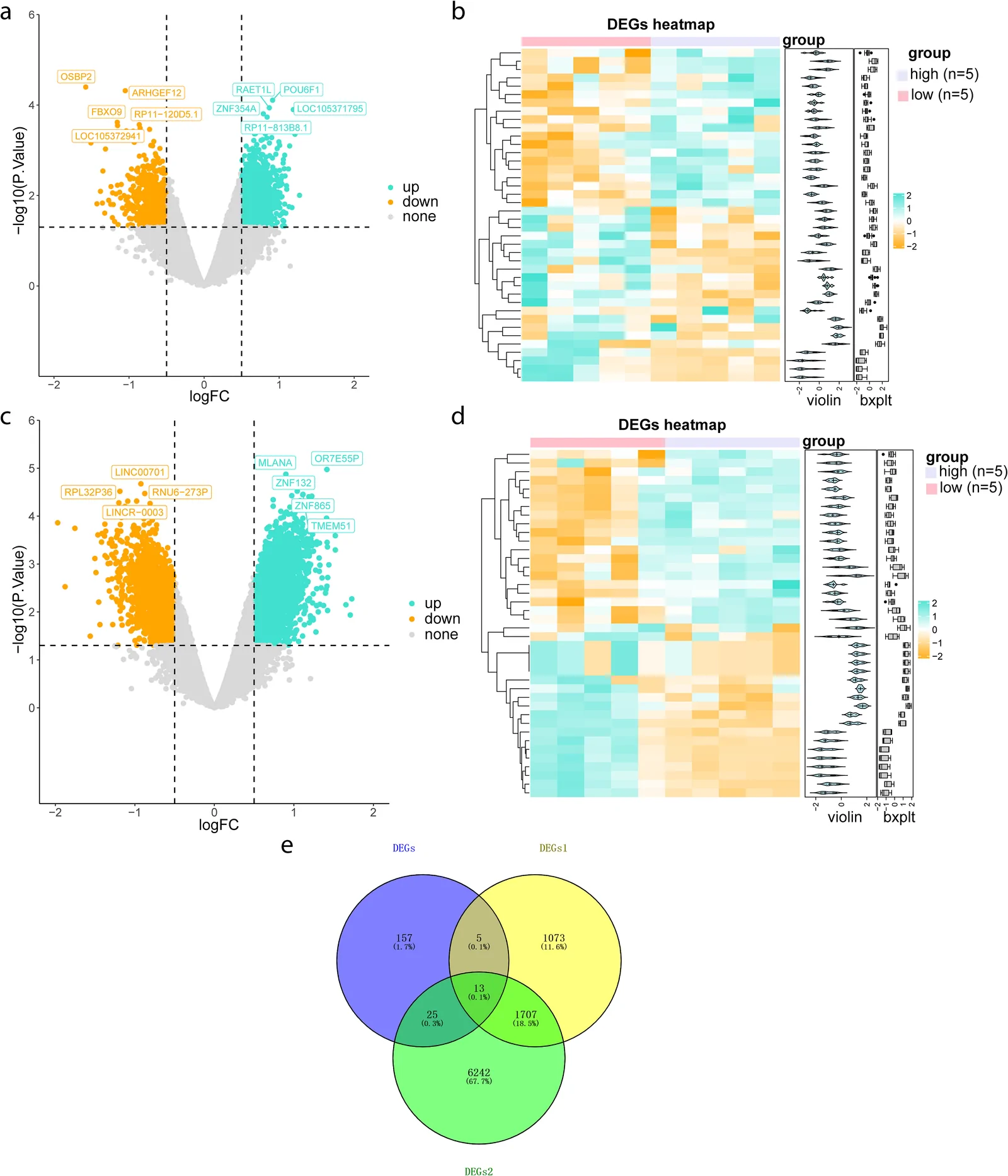
Screening of differentially expressed genes. (a) Volcano plot of DR-associated genes in GSE189005, where cyan indicates upregulation and orange indicates downregulation. (b) Heatmap of DR-associated genes in GSE189005, with higher expression shown in cyan and lower in orange. (c) Volcano plot of DR-associated genes in GSE221521, with cyan indicating upregulation and orange indicating downregulation. (d) Heatmap of DR-associated genes in GSE221521, with higher expression in cyan and lower in orange. (e) Venn diagram of upregulated DR-associated genes. (f) Venn diagram of downregulated DR-associated genes.

First, differential expression analysis identified 36,191 DR-associated genes in GSE189005, including 1,225 upregulated and 34,966 downregulated genes (Fig 2a–b). The large number of initial features arose from the extensive probe coverage of the microarray platform and the use of a relatively permissive nominal *p*-value threshold, which aimed to maximize screening sensitivity. To address concerns regarding the reliability of the study stemming from the high number of initial DR-associated genes, multiple rounds of intersection analysis and layered machine learning filtering were employed to reduce false positives. In GSE221521, 811 DR-associated genes were identified, comprising 519 upregulated and 292 downregulated genes (Fig 2c–d). Intersecting the genes exhibiting the same direction of differential expression yielded 200 consistently differentially expressed DR genes, which included 11 upregulated and 189 downregulated genes (Fig 2e–f).

**Fig 3:**
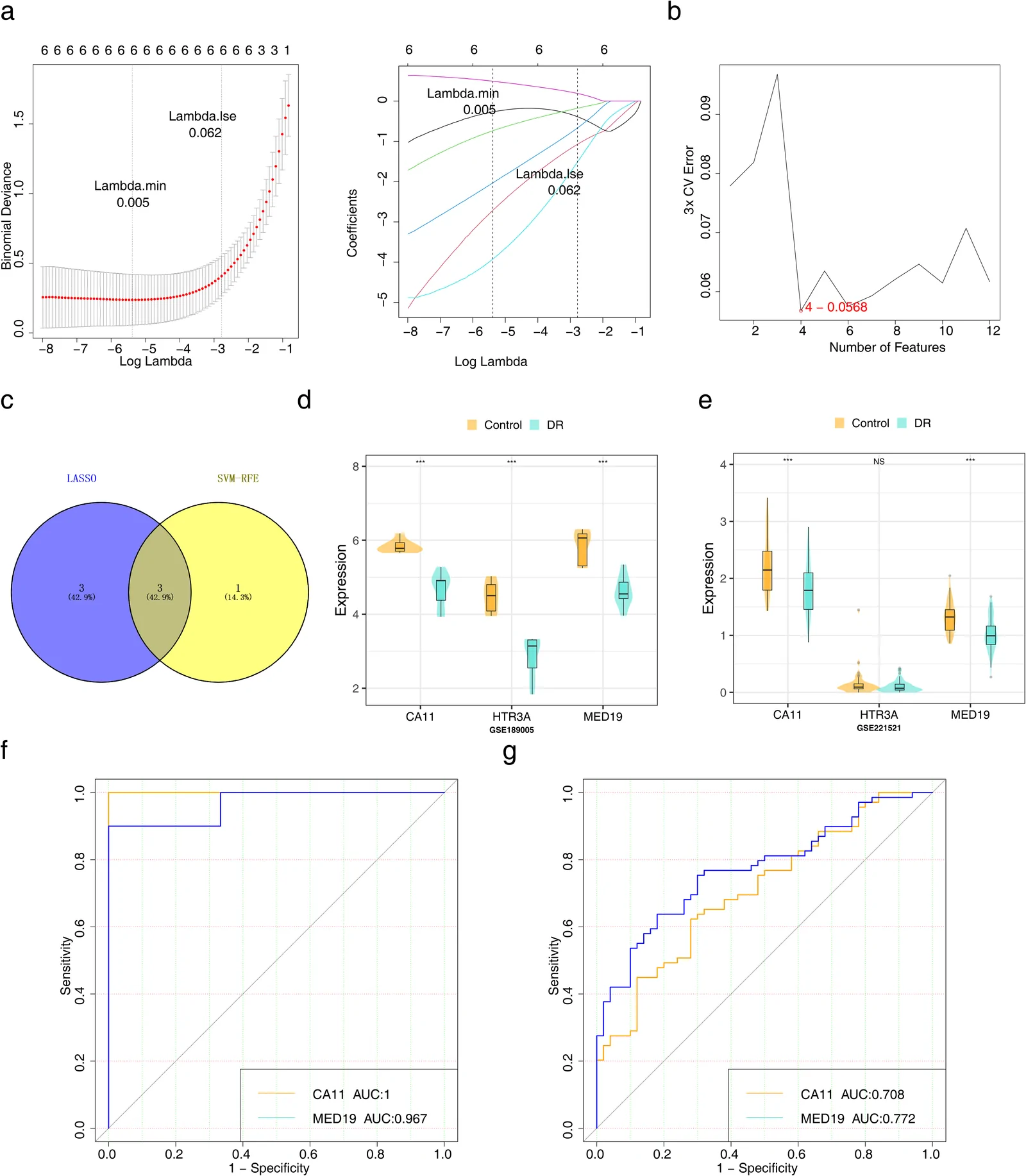
Screening of differentially expressed genes related to hypoxia and cuproptosis. (a) Volcano plot of hypoxia-score-associated genes between high- and low-score groups in GSE189005; cyan indicates upregulation and orange indicates downregulation. (b) Heatmap of hypoxia-score-associated genes between high- and low-score groups in GSE189005; higher expression shown in cyan and lower in orange. (c) Volcano plot of cuproptosis-score-associated genes between high- and low-score groups in GSE189005; cyan indicates upregulation and orange indicates downregulation. (d) Heatmap of cuproptosis-score-associated genes between high- and low-score groups in GSE189005; with higher expression in cyan and lower in orange. (e) Venn diagram of candidate genes.

Second, differential expression analysis between the high- and low-score groups identified 2,798 hypoxia-score-associated genes, which included 1,874 upregulated and 924 downregulated genes (Fig 3a–b). Similarly, 7,987 cuproptosis-score-associated genes were obtained for high- and low-score groups, including 4,982 upregulated and 3,005 downregulated genes (Fig 3c–d). Finally, the intersection of the 200 consistently differentially expressed DR genes with the two score-associated gene sets identified 13 candidate genes (Fig 3e).

### 3.2. MED19 and CA11 demonstrated superior diagnostic performance for DR

**Fig 4:**
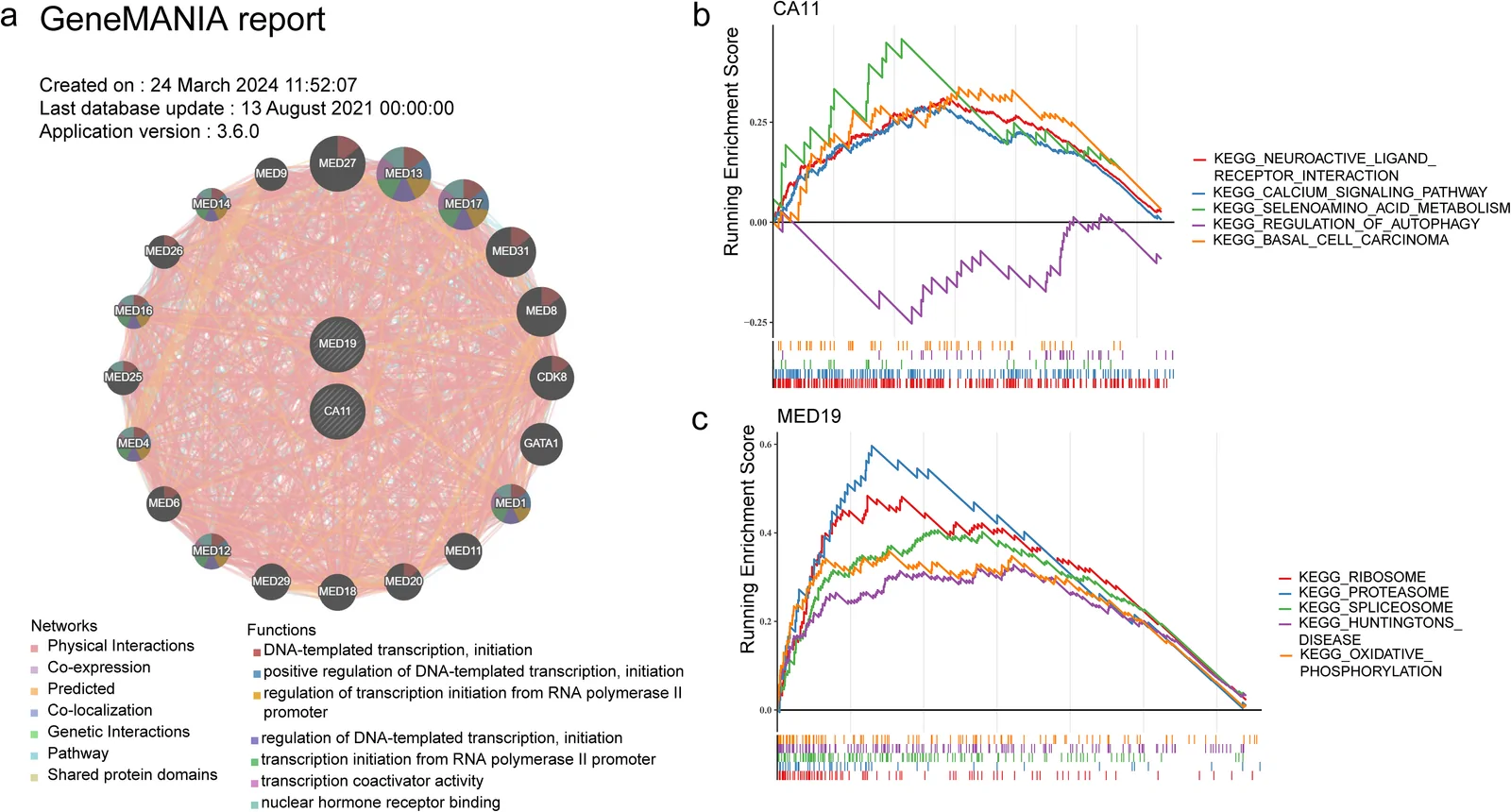
Screening of candidate biomarkers. (a) LASSO regression coefficient distribution and parameter plot. (b) SVM-RFE analysis showing generalization error versus number of features. (c) Venn diagram of genes selected by both machine learning methods.

Based on the 13 candidate genes, LASSO regression analysis selected 6 genes at lambda.min = 0.005 (Fig 4a), while SVM-RFE identified 4 genes at an error rate of 0.0568 (Fig 4b). The overlap of genes selected by both methods yielded three candidate biomarkers: HTR3A, MED19, and CA11 (Fig 4c).

**Fig 5:**
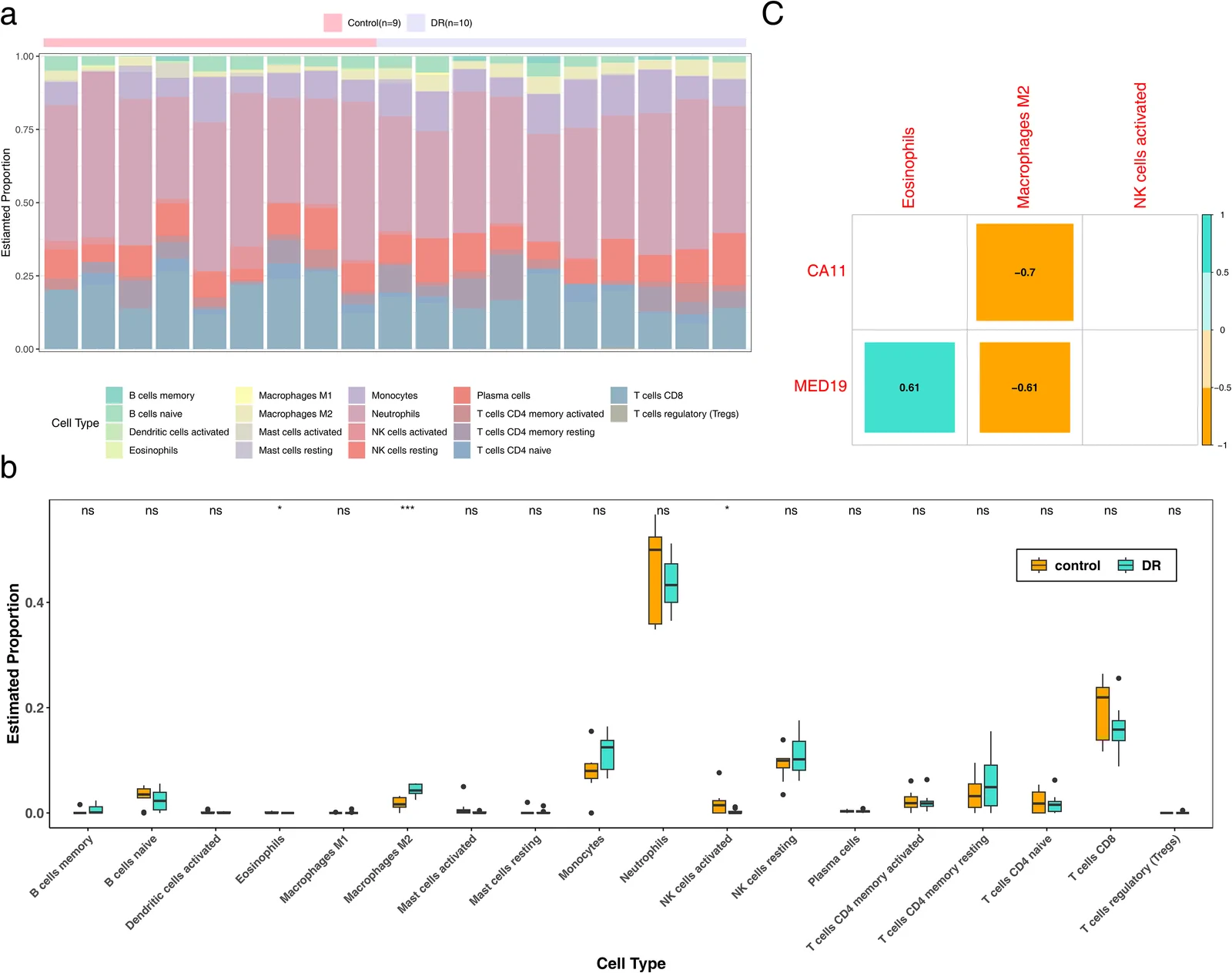
Screening of biomarkers. (a-b) Boxplots validating expression levels of candidate biomarkers in GSE189005 and GSE221521; ns indicates not significant, ***p < 0.001. (c-d) ROC curve validation of biomarkers in GSE189005 (c) and GSE221521 (d). (e) RT-qPCR validation of expression; significance: *p < 0.05.

MED19 and CA11 exhibited consistent expression patterns across GSE189005 and GSE221521, showing significantly reduced expression in DR samples in both datasets (P < 0.05) (Fig 5a-b). ROC analysis confirmed strong diagnostic performance, with AUC values for both MED19 and CA11 exceeding 0.7 in each dataset (Fig 5c-d). These genes remained significant after FDR correction, further supporting the robustness of the selection strategy. RT-qPCR validation corroborated the significant downregulation of MED19 and CA11 in DR samples (P < 0.05) (Fig 5e). Spearman correlation analysis in GSE189005 demonstrated positive associations of MED19 and CA11 with hypoxia markers (HIF1A, VEGFA) and cuproptosis markers (FDX1, LIAS) (|ρ| > 0.3, P < 0.05) (S2a Fig). These results support the association of expression levels between MED19/CA11 and hypoxia- and cuproptosis-related molecular patterns in DR, although they do not establish direct participation in specific pathways. Functional studies involving gene perturbation in retinal cells under high-glucose or hypoxic conditions, along with specific assays of cuproptosis, are necessary to clarify whether MED19 and CA11 play direct roles in these processes.

### 3.3. Functional analysis of MED19 and CA11 Revealed potential DR mechanisms

**Fig 6:**
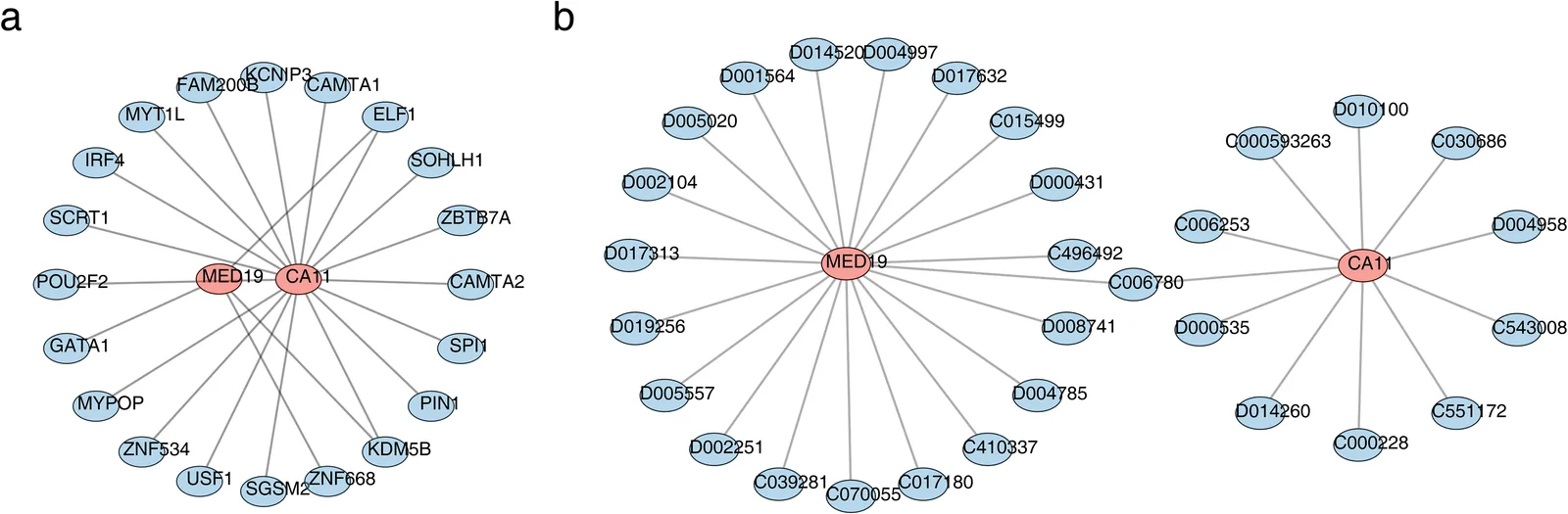
Analysis of biomarkers using GGI and GSEA. (a) GGI network of key genes. (b-c) GSEA enrichment analysis of MED19 (b) and CA11 (c).

Querying the GeneMINIA database identified 20 biomarker-associated genes, leading to the construction of a GGI network that included interactions such as MED13–MED19 and CDK8–CA11 (Fig 6a). Co-associated functions included “DNA-templated transcription, initiation” and “nuclear hormone receptor binding.”

GSEA revealed significant enrichment of MED19 in 73 KEGG pathways and CA11 in 12 pathways. The top five pathways associated with MED19 were “proteasome,” “spliceosome,” “ribosome,” “Huntington's disease,” and “oxidative phosphorylation” (Fig 6b). For CA11, the top five pathways included “selenoamino acid metabolism,” “neuroactive ligand–receptor interaction,” “calcium signaling pathway,” “regulation of autophagy,” and “basal cell carcinoma” (Fig 6c).

### 3.4. M2 macrophages showed significant negative association with biomarkers

**Fig 7:**
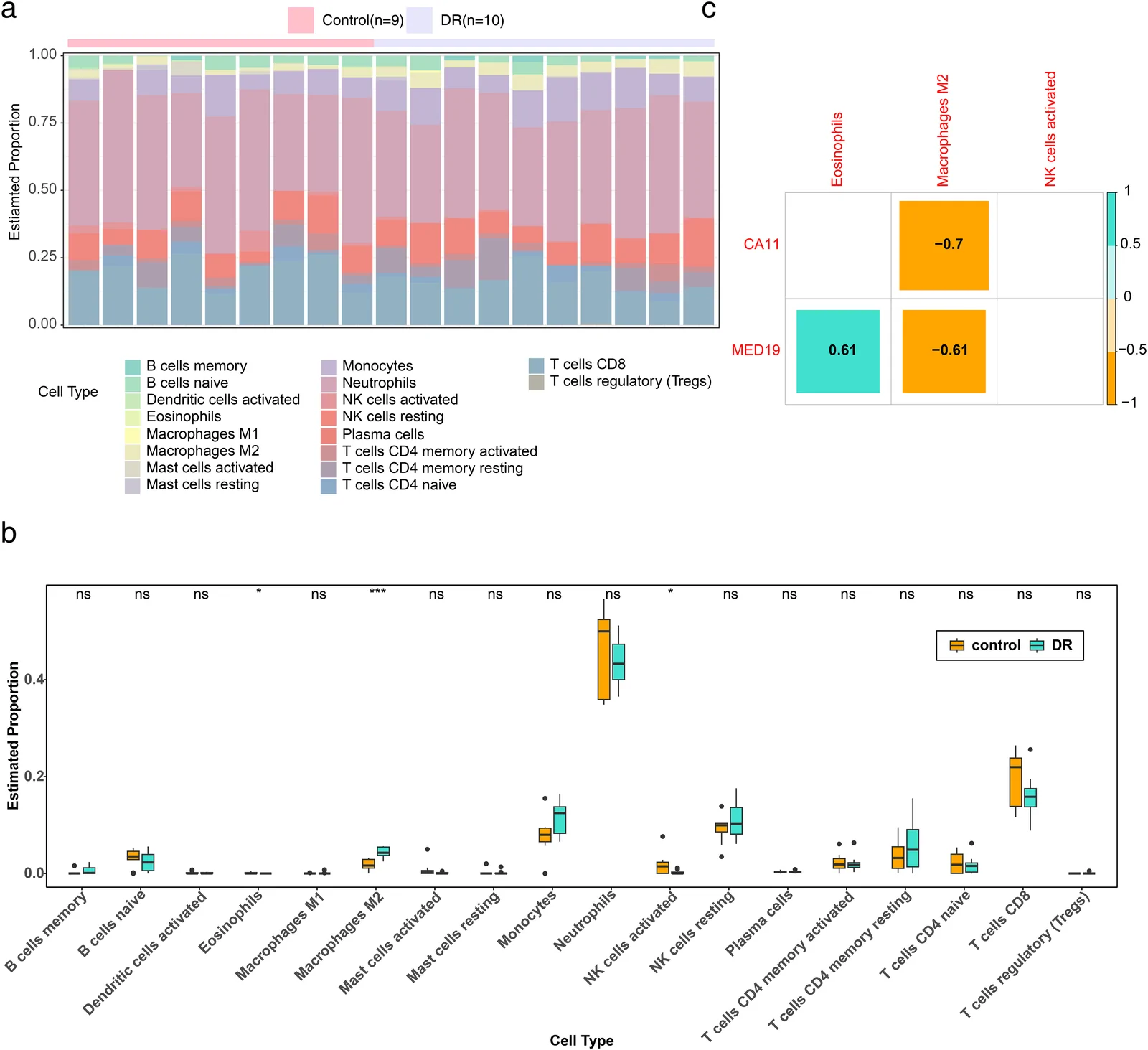
Immune infiltration analysis. (a) Heatmap of immune infiltration abundance; colors from blue to red indicate increasing abundance. The vertical axis represents immune cell types. (b) Boxplots of differentially expressed immune cells; ns indicates not significant, *p < 0.05, ***p < 0.001. (c) Heatmap of correlations among differentially expressed immune cells; green indicates positive correlation, orange indicates negative correlation, with darker colors representing stronger correlations.

After excluding immune cells with zero infiltration abundance, the remaining immune cell proportions were analyzed (Fig 7a). Three immune cell populations—eosinophils, M2 macrophages, and activated natural killer (NK) cells—demonstrated significant differences in abundance between DR and control samples in GSE189005 (Fig 7b). Specifically, eosinophils and activated NK cells were reduced in DR samples, while M2 macrophages were elevated (Fig 7b). Spearman correlation analysis revealed significant negative correlations between M2 macrophages and both MED19 (r = −0.61, P < 0.05) and CA11 (r = −0.70, P < 0.05). Conversely, eosinophil levels were positively associated with MED19 (r = 0.61, P = 0.023), while activated NK cells showed no significant correlation with either biomarker (Fig 7c). Further analysis indicated that MED19 and CA11 were positively correlated with immune-regulatory molecules, suggesting potential roles in M2 recruitment/polarization (CCL2/CCL22) and immune checkpoint–mediated anti-inflammatory responses (PDCD1, CD274, CTLA4, LAG3, HAVCR2, IL10) (S2b Fig).

### 3.5. Regulatory Network Analysis Explored Potential DR Mechanisms

ChEA3 database queries predicted 255 TFs targeting the biomarkers. The top 20 TFs were utilized to construct a TF-mRNA regulatory network, consisting of 22 nodes and 22 edges (S3a Fig), with notable interactions such as ELF1–MED19 and KDM5B–CA11. Based on these two biomarkers, 30 candidate compounds/interventions were predicted, and a drug-biomarker network was established, comprising 32 nodes and 31 edges (S3b Fig). Only drug IDs are depicted in the figure, while corresponding drug names are provided in S3 and S4 Tables. Bisphenol A (BPA, C006780) emerged as a compound co-targeting MED19 and CA11 (S3b Fig). To differentiate potentially safer candidates from those associated with known toxicological concerns, all candidates were classified according to endocrine-disrupting potential, other documented toxicities, and ophthalmology-related research evidence. Five candidates received Tier 1 classification, while two candidates were assigned to Tier 2. Twenty-two compounds with known or suspected endocrine-disrupting properties or other documented toxicities were classified as Tier 3. “Environmental Pollutants” was not tiered, as it represents a broad exposure category rather than a structurally defined compound (S5 Table). A prioritized candidate compound/intervention–biomarker association network was constructed using Tier 1 and Tier 2 candidates along with BPA (C006780), comprising 10 nodes and 9 edges (S3c Fig). MED19 was linked to fenretinide, K 7174, schisandrin B, and BPA, while CA11 was associated with oxygen, pirinixic acid, acetamide, afuresertib, and BPA. BPA was the only compound associated with both MED19 and CA11. However, due to BPA's classification as a known endocrine disruptor, it was categorized as Tier 3 and included in the network solely to illustrate its shared database association, rather than indicating therapeutic priority.

## 4. Discussion

DR represents a severe microvascular complication of diabetes mellitus, characterized by a multifactorial pathogenesis that includes oxygen deprivation [22]. Emerging evidence also implicates cuproptosis in the development of diabetes [23]; however, its potential role in DR secondary to diabetes remains largely unexplored. Systematic analyses of HRGs and CRGs in DR are also lacking. This study identified MED19 and CA11 as potential biomarkers for DR using bioinformatics approaches, providing novel insights into DR pathogenesis and offering perspectives for early diagnosis and targeted interventions.

MED19 and CA11 were identified as candidate DR biomarkers through differential expression analysis and machine learning. MED19, a mediator complex subunit, regulates transcription and modulates RNA polymerase II activity [24], participating in processes such as transcriptional control, cellular proliferation, and apoptosis [25]. MED19 has been implicated in various malignancies, including gastric, colorectal, and bladder cancers. Inhibition of MED19 in non-small cell lung cancer cells confers resistance to cisplatin and paclitaxel [26], while in hepatocellular carcinoma, MED19 knockdown suppresses proliferation, induces G0/G1 arrest, and inhibits tumor development [26]. In tongue carcinoma, MED19 knockdown results in G1 phase arrest, reduced proliferation, migration, and tumorigenicity [27]. In osteosarcoma, MED19 is associated with proliferation and metastasis, potentially via AKT-mediated cyclin regulation, though the precise mechanisms remain to be elucidated [26]. Although not directly studied in DR, MED19's fundamental role in transcriptional regulation suggests it may similarly influence retinal cell function.

CA11, a member of the carbonic anhydrase family, primarily regulates extracellular pH and cellular proliferation [28]. Carbonic anhydrases maintain systemic acid-base homeostasis by modulating intra- and extracellular pH [29]. Altered acid-base balance can negatively impact retinal health, particularly in conditions such as DR and macular degeneration [30]. Therefore, CA11 may contribute to retinal homeostasis by regulating extracellular fluid and pH in retinal cells, although its specific role in DR has yet to be reported.

The enrichment of MED19 in the “Proteasome,” “Spliceosome,” and “Ribosome” pathways highlights its central role in protein degradation and synthesis [31]. Impaired proteasome activity is associated with diabetes progression and complications, including DR, as hyperglycemia may reduce proteasome function, triggering endoplasmic reticulum stress, cellular dysfunction, and apoptosis [32]. Dysfunction of the ribosome similarly affects protein synthesis and is linked to diabetes-related pathology [31]. These findings suggest MED19 may mediate DR pathogenesis by regulating proteasome, spliceosome, and ribosome pathways, thereby influencing retinal cellular function, stress responses, and survival under hyperglycemic conditions. Further studies are required to validate MED19's specific role in DR progression and its therapeutic potential.

CA11, a member of the carbonic anhydrase family, is involved in regulating extracellular pH and cellular proliferation. KEGG pathway enrichment analysis indicated CA11's involvement in selenoamino acid metabolism, neuroactive ligand–receptor interactions, calcium signaling, autophagy regulation, and basal cell carcinoma pathways. The selenoamino acid metabolism pathway plays a critical role in antioxidant defense, suggesting that CA11 may modulate oxidative stress in DR through the regulation of selenoprotein expression or activity [33]. Oxidative stress promotes cellular damage via the excessive accumulation of free radicals, mitochondrial dysfunction, and lipid peroxidation, while bioactive compounds with antioxidant activity may mitigate these pathological changes [34]. Although substantial research highlights the significance of oxidative stress-related pathways in the context of DR, it does not directly demonstrate CA11's capacity to regulate oxidative damage. CA11's enrichment in the neuroactive ligand–receptor interaction pathway suggests potential involvement in neurovascular injury in DR by influencing neurotransmission and cellular signaling [33]. Additionally, through the calcium signaling pathway, CA11 may impact both cellular signaling and oxidative stress by regulating calcium homeostasis [35]. Autophagy-related pathways are essential for maintaining cellular homeostasis, and CA11 may play a role in cellular damage repair and metabolic regulation in DR by modulating autophagy. The basal cell carcinoma pathways, which regulate cellular proliferation and apoptosis, imply that CA11 could influence DR pathogenesis similarly.

Immune infiltration analysis revealed significant differences between DR and control samples concerning eosinophils, M2 macrophages, and activated NK cells. MED19 and CA11 exhibited significant negative correlations with M2 macrophages, indicating that reduced expression of these biomarkers may be associated with impaired M2 macrophage functions in tissue regeneration, damaged cell clearance, and immune modulation. This inverse relationship could contribute to retinal pathological changes by disrupting tissue repair and immune balance. Further, MED19 and CA11 were positively correlated with CCL2/CCL22 (M2 recruitment/polarization), IL10 (anti-inflammatory M2 marker), and immune checkpoints (PD-1/PD-L1, CTLA4, LAG3, TIM-3), consistent with roles in M2 macrophage-related immune regulation [36]. Their downregulation in DR may therefore reflect reduced M2 activity mediated by chemokine and immune checkpoint pathways, contributing to retinal immune imbalance. Eosinophils may exacerbate diabetic complications, including DR, through the release of pro-inflammatory factors [37], while activated NK cells could intensify retinal damage through cytotoxicity or pro-inflammatory signaling [38]. Collectively, MED19 and CA11 are closely linked to the DR immune microenvironment and may participate in pro-inflammatory or pro-fibrotic processes during disease progression. These observations provide insights into the immune regulatory networks in DR and potential avenues for targeted therapies.

To further explore the potential upstream regulatory mechanisms of MED19 and CA11, the ChEA3-predicted regulatory network was classified based on correlations between candidate regulatory factors and hypoxia/HIF signaling, as well as copper-mediated cell death. Among the predicted regulatory factors, ZBTB7A, GATA1, USF1, PIN1, and KDM5B emerged as hypoxia/HIF-associated regulators, with KDM5B also identified as an epigenetic regulatory factor linked to copper-mediated cell death. These associations suggest that alterations in the activity of regulatory factors related to hypoxia or copper homeostasis may contribute to the downregulation of MED19 and CA11 in DR. In a hypoxic microenvironment, changes in transcriptional or epigenetic regulatory activity could influence the transcription of these candidate markers. However, since this regulatory network is derived from database predictions, it cannot substantiate that the aforementioned regulatory factors directly bind to the MED19 or CA11 promoter regions, nor can it establish causal regulatory relationships. Notably, typical HIF family members did not appear among the top-ranked predictions in ChEA3; therefore, they were excluded from the network in the absence of predictive evidence. Future studies are required to validate this potential regulatory pattern through promoter binding site analysis, chromatin immunoprecipitation, luciferase reporter assays, and gene interference experiments under high-glucose or hypoxic conditions.

Drug prediction and analysis identified 30 candidate compounds, including schisandrin B, pirinixic acid, and fenretinide. Schisandrin B has gained attention due to recent ophthalmic studies, demonstrating its ability to alleviate oxidative stress, inflammatory responses, and retinal tissue damage induced by a high-sugar diet in zebrafish, while simultaneously reducing reactive oxygen species and malondialdehyde levels in the retina and increasing superoxide dismutase activity [39]. Additionally, schisandrin B can enhance glucose metabolism and mitigate pancreatic tissue damage in type 2 diabetes models by activating the GLP-1R/cAMP/PKA pathway, thereby supporting its potential use in addressing diabetes-related complications [40]. Nonetheless, further experimental validation is needed to ascertain whether schisandrin B can regulate MED19 and to evaluate its efficacy and safety in mammalian models of DR. Pirinixic acid, also known as WY-14643, has shown potential in increasing the survival rate of retinal ganglion cells in an *in vitro* model of injured mouse retina, indicating possible retinal neuroprotective effects [41]. However, the regulation of CA11 expression by pirinixic acid and its effects on diabetes-induced retinal microvascular damage remain unclear. Fenretinide, investigated in retinal disease research due to its influence on retinoic acid metabolism, was previously noted in a clinical study for causing a decline in dark adaptation; however, these changes typically resolve after discontinuation of the drug or supplementation with vitamin A [42]. Overall, the candidate compound–marker associations identified in this study are hypothesis-generating findings. Their regulatory mechanisms, effects on MED19 and CA11 expression, retinal bioavailability, ocular toxicity, and therapeutic efficacy require further validation in retinal cells and experimental models of DR.

This study successfully identified two key genes associated with DR, MED19 and CA11, through multi-omics analysis. Bioinformatics analyses indicated significantly reduced expression levels of these genes in DR samples, alongside strong diagnostic performance. Both genes exhibited negative correlations with M2 macrophages and involvement in molecular regulatory networks, establishing a foundation for early diagnosis and targeted therapy for DR.

Several limitations exist in this study. First, as a bioinformatics analysis, the findings necessitate further experimental validation, including knockout or animal model studies, to elucidate specific molecular mechanisms. The observed correlations with hypoxia and cuproptosis markers are suggestive and require *in vitro* confirmation to establish causality. Second, the public datasets and clinical validation cohort were relatively small. The RT-qPCR cohort included only five patients with DR and five controls, resulting in limited statistical power and an increased risk of Type II error; as a consequence, potentially meaningful expression differences may not have been detected. Additionally, the small sample size hindered reliable adjustments for potential confounders, including age, sex, diabetes duration, glycemic control, medication use, and DR stage. Moreover, all five patients with DR were classified as stage IV, limiting comparisons across various disease stages, such as non-proliferative and PDR, and thereby constraining the cohort's ability to represent the clinical heterogeneity of real-world patients. As a result, the RT-qPCR findings should be seen as preliminary expression-level evidence rather than definitive clinical validation. Future studies should validate MED19 and CA11 in considerably larger, adequately powered, multicenter peripheral-blood cohorts, ideally including several hundred participants with comprehensive clinical information and predefined stratification according to diabetes duration and DR stage. Furthermore, since DR arises within the context of broader systemic metabolic and inflammatory abnormalities, future research may also investigate the potential role of gut-derived metabolic signals. Recent studies have indicated that gut microbiota remodeling, metabolic reprogramming of the microbiota, and the effects of unabsorbed bioactive components via microbiota-dependent pathways may influence the host's metabolic and immune homeostasis [43,44]. However, this study did not examine dietary interventions, gut microbiota composition, or microbial metabolites, making it impossible to infer a direct relationship between these mechanisms and MED19 or CA11 based on the current results. Future studies integrating peripheral blood transcriptomic data alongside gut microbiota and microbial metabolomic data may help elucidate any associations between gut-derived metabolic changes and the molecular and immunological alterations linked to DR.

## 5. Conclusions

In conclusion, MED19 and CA11 emerge as key diagnostic biomarkers for DR, characterized by marked downregulation and high diagnostic accuracy. Their involvement in critical pathways, such as proteasome function and autophagy, along with correlations with immune infiltration, highlights their roles in DR pathogenesis related to hypoxia and cuproptosis. These findings offer novel insights for developing diagnostic strategies and targeted therapies.

## Supporting information

S1 Table Clinical information of enrolled subjects. (XLSX)

S2 Table Primer sequences of RT-qPCR. (XLSX)

S3 Table Drug prediction results of CA11. (XLSX)

S4 Table Drug prediction results of MED19. (CSV)

S5 Table Toxicity classification and ophthalmology relevance of 30 predicted compounds. (XLSX)

S1 Fig Principal component analysis (PCA) of GSE189005 and GSE221521 datasets. (A) PCA of GSE189005 microarray samples (DR vs. control). (B) PCA of GSE221521 RNA-seq samples (DR vs. control). Both datasets showed clear within-group clustering without apparent systematic batch effects. Each point represents an individual sample, with colors and symbols indicating the diabetic retinopathy (DR) and control groups. Ellipses represent the 95% confidence regions for each group. The horizontal and vertical axes indicate the first and second principal components, respectively, and the percentages in parentheses represent the proportion of total variance explained by each principal component.
(TIF)

S2 Fig Correlation heatmaps of MED19 and CA11 with hypoxia/cuproptosis-related markers and immune-regulatory molecules. (A) Spearman correlation analysis between MED19/CA11 and hypoxia markers (HIF1A and VEGFA) and cuproptosis markers (FDX1 and LIAS) in GSE189005. The horizontal axis represents hypoxia- and cuproptosis-related markers, while the vertical axis represents the analyzed biomarkers. (B) Spearman correlation analysis between MED19/CA11 and immune-regulatory molecules, including cytokine (IL10), chemokines (CCL2 and CCL22), and immune checkpoint molecules (PDCD1, CTLA4, LAG3, HAVCR2, and CD274). The horizontal axis represents immune-regulatory molecules, while the vertical axis represents the analyzed biomarkers. Color intensity indicates the strength of correlation. Statistical significance is indicated as follows: *P < 0.05, **P < 0.01, ***P < 0.001, and ****P < 0.0001.
(TIF)

S3 Fig Regulatory network analysis. (a) TF–mRNA regulatory network. Orange nodes represent hypoxia/HIF-related regulators, green nodes represent cuproptosis-related regulators, and orange nodes with green borders represent regulators associated with both categories. Blue nodes represent other predicted regulators, while red nodes represent MED19 and CA11. (B) Candidate compound–biomarker network. Blue nodes represent predicted compounds, and red nodes represent biomarkers. (C) Prioritized database-derived compound–biomarker association network. Red hexagons represent the candidate biomarkers MED19 and CA11. Green and yellow circles represent Tier 1 and Tier 2 candidate compounds or interventions, respectively, whereas gray circles represent non-prioritized compounds with potential toxicological or endocrine-disrupting concerns. Solid edges indicate database-derived compound–biomarker associations. Dashed edges indicate the shared database association of bisphenol A (BPA) with MED19 and CA11. BPA was retained only to illustrate this shared association and was not considered a prioritized therapeutic candidate.
(TIF)
